# Verifying learning success: assessment and certification of student ultrasound education

**DOI:** 10.1186/s12909-025-07469-0

**Published:** 2025-06-19

**Authors:** Ricarda Neubauer, Florian Recker, Johannes Weimer, Christian Jenssen, Dieter Nürnberg, Thomas Karlas, Susan Campbell Westerway, Johannes P. Ruppert, Nils Daum, Anna Gschmack, Yi Dong, Kathleen Möller, Nasenien Nourkami-Tutdibi, Gregor Barth, Roxana Şirli, Claudia Lucius, Michael Prats, Sophie-Luise Sänger, Michael Keil, Constantinos Zervides, Beatrice Hoffmann, Christoph F. Dietrich

**Affiliations:** 1 Department of Obstetrics and Prenatal Medicine, University Hospital Bonn, Bonn, Germany; 2 Rudolf-Frey Teaching Department, Medical Center of the Johannes Gutenberg University Mainz, Mainz, Germany; 3 Department of Internal Medicine, Krankenhaus Märkisch Oderland, Strausberg, Germany; 4 Brandenburg Institute for Clinical Ultrasound, Brandenburg Medical School Theodor Fontane, Neuruppin, Germany; 5 Faculty of Health Science Brandenburg, Brandenburg Medical School Theodor Fontane, Neuruppin, Germany; 6 Department of Internal Medicine, Division of Gastroenterology, Leipzig University Medical Center, Leipzig, Germany; 7 School of Dentistry & Health Sciences, Charles Sturt University Australia, Wagga Wagga, Australia; 8 Justus-Liebig-University, Giessen, Germany; 9 Anesthesiology and Intensive Care Medicine, Charité Universitätsmedizin Berlin, Berlin, Germany; 10 Department of Ultrasound, Xinhua Hospital Affiliated to Shanghai Jiaotong University School of Medicine, Shanghai, China; 11 Gastroenterology, Sana Hospital Lichtenberg, Berlin, Germany; 12 Hospital for General Paediatrics and Neonatology, Saarland University Medical Center, Homburg/Saar, Germany; 13 Department of Hematology, Oncology and Palliative Care, University Hospital Brandenburg, Brandenburg Medical School Theodor Fontane, Brandenburg, Germany; 14 Department of Gastroenterology and Hepatology, Center for Advanced Research in Gastroenterology and Hepatology, “Victor Babeș” University of Medicine and Pharmacy, Timișoara, Romania; 15 Outpatient Department of Gastroenterology, IBD center, Policlinic Helios Klinikum Buch, Berlin, Germany; 16 Department of Emergency Medicine, Division of Ultrasound, The Ohio State University Wexner Medical Center, Columbus, OH USA; 17 Faculty of Medicine, Technische Universität Dresden, Dresden, Germany; 18 Johann Wolfgang Goethe University, Frankfurt am Main, Germany; 19 CZMH Medical Physics and Dosimetry Services LTD, Limassol, Cyprus; 20 Department of Emergency Medicine, Beth Israel Deaconess Medical Center, Harvard Medical School, Boston, USA

**Keywords:** Undergraduate ultrasound education, Assessment, Certification

## Abstract

Student ultrasound education (SUSE) is currently composed of heterogeneous curricular training formats, and new approaches are continually being explored to enhance undergraduate ultrasound training. Based on a literature review, this report aims to analyze and compare different forms and methods of assessment of acquired skills in ultrasound training. Therefore, the advantages and disadvantages of assessment formats and certification systems used in SUSE were discussed collaboratively between medical students, postgraduate physicians, and medical didactics experts. Ultrasound competency should be tested using structured examination formats that are objective and standardized. In addition to cognitive skills, the examination format should cover translational and behavioral components. Self-assessments and evaluations provide additional valuable perspectives. Certification systems can contribute to quality assurance by externally ensuring the achievement of milestones in SUSE. They also have the potential to support the necessary standardization of undergraduate ultrasound teaching by aligning the curricular learning objectives with qualifications to be achieved.

## Introduction

Student ultrasound education (SUSE) is a dynamically evolving field that plays a critical role in equipping medical students with practical imaging skills essential for modern diagnostics [1, 2]. The World Federation for Ultrasound in Medicine and Biology (WFUMB) recommends early implementation of ultrasound into medical curricula to familiarize students with this essential modality at the outset of their training [3, 4]. However, significant challenges remain in ensuring the consistency and quality of SUSE implementation and assessment.

Effective assessment of ultrasound skills is critical for several reasons. First, it ensures that students achieve a minimum standard of competence in performing and interpreting ultrasound examinations, which is essential for patient safety and diagnostic accuracy. Proper assessment reduces the risk of false-negative findings caused by insufficient examination or overdiagnosis resulting from misinterpretation. Second, consistent evaluation helps to identify gaps in student learning, enabling targeted interventions to improve their skillset. Finally, standardized assessment formats provide a benchmark for comparing competencies across different institutions, ensuring equitable training outcomes.

Despite the importance of skill acquisition and assessment, current approaches in SUSE vary widely. It is likely that the existence of obstacles to the implementation of a structured SUSE curriculum is a contributing factor. One such obstacle, as identified in a survey conducted by Recker et al. among German medical students, is insufficient time dedicated to ultrasound training [5]. Furthermore, assessment methods range from written exams to OSCEs and non-standardized practical evaluations, which hinders the comparability of measured competencies [6]. This heterogeneity poses challenges to both educators and students, underscoring the need for standardized assessment frameworks and certification systems.

This article aims to address these challenges by discussing and comparing various assessment forms in SUSE and evaluating their potential impact on educational outcomes. Furthermore, it explores the advantages and disadvantages of introducing certification systems as a means to standardize and improve ultrasound education in medical training. By addressing these issues, the study seeks to bridge existing gaps and contribute to the development of more effective and consistent approaches to SUSE assessment.

## Development of competence and appropriate assessment

Assessments are a key tool for quality control and for gathering evidence of knowledge, skills, and competence. Serial tests can also provide insight into knowledge retention over time. Furthermore, an upcoming exam can increase the students’ motivation to practice and reinforce what has been learned previously [7]. Moreover, students also receive direct feedback on their skills through tests. According to Hattie’s comprehensive meta-analysis of educational influences, feedback and the ability to self-assess have high effect sizes significantly promoting learning success [8]. Applying these findings to medical students suggests that constructive feedback enhances the learning process. It is worth noting that feedback in the form of undifferentiated grading is probably the least effective option of different feedback methods as it provides minimal guidance for improvement. Nevertheless, differentiated feedback can potentially enable students to improve their ability to reflect on their own level of performance, strengthen their intrinsic motivation and experience self-efficacy by recognizing the progress they are making towards their individual goals. By identifying strengths as well as areas for improvement, students can engage more intensively with the learning content.

**Fig. 1:**
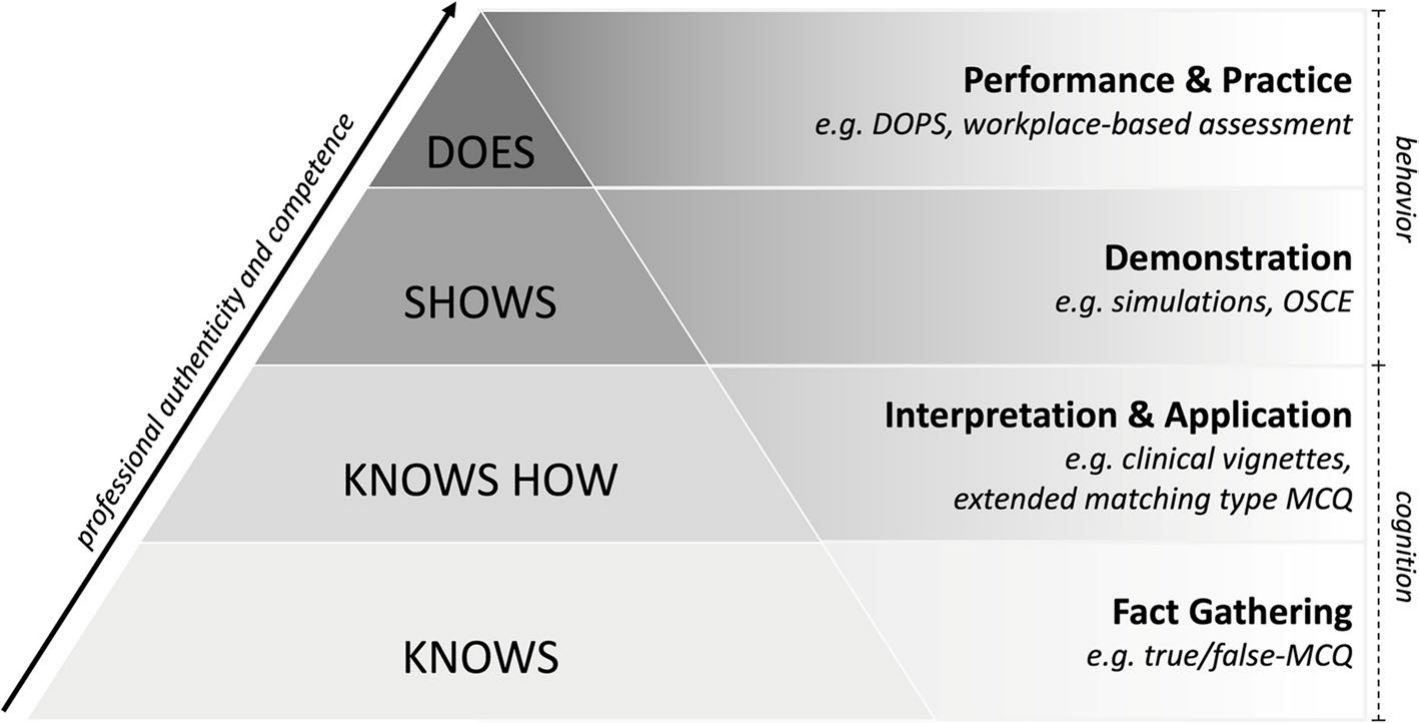
Miller's assessment and clinical competence pyramid shows the progression from fundamental cognitive learning to expert behavioral learning [9]. *MCQ: Multiple Choice Questionnaire; OSCE: Objective Structured Clinical Examination, DOPS: direct observation of procedural skills*

Assessment should be objective, reliable, and valid, with results presented either summatively or formatively. According to Miller’s pyramid, different test formats align with various competence levels (see Fig. 1) [9]. The foundation begins with acquiring knowledge in ultrasound physics and techniques, measurable by self-assessment or objective tests like single or multiple choice questionnaires (MCQs) or oral exams. Next, knowledge application in clinical contexts is evaluated through complex MCQs related to case vignettes. At the third level, behavioral changes in the use of ultrasound are evaluated as a complex and dynamic capability that combines image acquisition, optimization and simultaneous interpretation of real-time images as well as the adaptation of the examination accordingly. During an *Objective Structured Clinical Examination (OSCE)*, the examinee passes through various examination stations in a simulated clinical environment using standardized checklists on skills and context-related behavior [10, 11]. Finally, the top of the pyramid represents the controlled application of acquired ultrasound skills in a clinical context. The *Direct Observation of Procedural Skills (DOPS)* is a workplace-based test tool developed to evaluate skills in their clinical context. The assessment can be formative or summative [10, 12]. As an assessment method involving patients, it requires a certain level of clinical experience and therefore might be less suitable in undergraduate settings. For this purpose, simulations or acting patients may be a suitable alternative. Further assessment methods evaluate the quality of the acquired ultrasound images, for example, using standardized rating systems, such as the Brightness mode quality ultrasound imaging examination technique (B-QUIET) [13]. In the following, various approaches in measuring skills and competencies are presented and possible advantages and disadvantages of their application are discussed.

## Purposes und timing of assessments

An examination can have different purposes. While it can assist in measuring students’ skills to grade their competence, examination results can also be used to conclude the effectiveness of the didactic approach based on the learning success achieved by participating students. To identify appropriate test tools and timing, the aim of the assessment should be clarified in advance. When evaluating a training, a baseline test of pre-existing skills should be performed before the teaching intervention. The obtained results can then be compared with the score after the ultrasound training. Furthermore, it is essential to differentiate between the subjective perception of organizers and participants and an objective assessment of achieved learning objectives. Evaluation forms provide only subjective parameters and, therefore, cannot independently prove the effectiveness of a teaching intervention. Nevertheless, the views, opinions, and perspectives of different people involved, such as teachers, students, and additional supporting staff, are essential to effectively rate a training format. These valuable insights should be considered for further development of educational programs according to the needs of both instructors and learners.

As with any form of knowledge or practical skill, acquired ultrasound skills will decrease over time if not continually reinforced by repeated practice [14]. With ultrasound being an operator-dependent imaging tool, it is imperative to consider this aspect. Therefore, the minimum number of annually performed ultrasound examinations and regularly attended training events occupies a central role in the certification systems of ultrasound societies. However, while clinicians rather have the opportunity to apply their ultrasound skills continuously during clinical practice, medical students often lack sufficient access to ultrasound equipment, patients and devoted curricular time to maintain acquired ultrasound skills. Instead, a continuous training process as in the form of a longitudinal curriculum would enable students to sustainably acquire ultrasound skills [15–18]. To date, only few studies examine the long-time retention of ultrasound skills to identify the time when further training should take place [2, 19]. Additionally, due to the varying complexity of ultrasound examinations, the appropriate timing in the curriculum can differ from one type of examination to another as well as according to different curricular structures [19]. Nevertheless, a general assessment of acquired ultrasound skills should be performed before their clinical application (e.g. during the last year of medical school) [17].

## Comparison and discussion of assessment methods

### No assessment of acquired competence

The organization and conduction of ultrasound assessments are time-consuming, and resources could be saved if no assessment or control of learning outcomes were conducted. From a student perspective, the absence of a graded assessment can relieve stress for medical students [20]. Furthermore, the intrinsic motivation of the students can be strengthened in favor of personal ambition to acquire as many ultrasound skills as possible for their future career instead of the exam being the primary motivator. Even without structured protocols, experienced instructors may have a good insight of individual students’ skill development and evolving competence based on questions, discussions, and the supervision of independent scanning during training sessions [21]. Individualized feedback by the supervisor can set individual learning priorities and highlight strengths and weaknesses. However, implementing this on a broad scale may not be feasible due to the significant personnel and time requirements involved.

On the other hand, if the learning success is not measured, the effectiveness of the ultrasound training, including the teaching methods and tools used, cannot be objectively evaluated. However, the evaluation of any educational format is critical for its further enhancement. Furthermore, checking the students’ learning success can have a motivating effect on the students by receiving feedback on the acquired skills and the comparison of intra- and interindividual progress. Ultimately, certificates that are intended to ensure uniform quality must be evaluated according to an objective standard. In the absence of an assessment, the extent to which acquired skills have been sufficiently acquired remains less determinate.

### Self-assessment using questionnaires

Digital questionnaires for self-assessment are easily implemented and associated with lower personnel costs and lower financial commitment as they can be performed at any time and place. Self-assessment can increase one’s motivation to develop skills because it relates current skills and knowledge to desired future goals [10]. Combining self-assessment tools with objective parameters can help students to better reflect on the extent and limits of their skills [22]. However, using exclusively self-assessment questionnaires, only the subjective perceptions of students are measured, which can be biased by personal factors, such as individual effort and emotions, and lacks objective metrics for evaluating learning outcomes.

A study by Steinmetz et al. revealed that students estimated their ultrasound skills on average 68% better in self-assessment than they would have been evaluated by an external examiner [23]. On the other hand, results of three meta-analysis conducted showed that students are moderately able to self-assess their general medical knowledge and skills, with accuracy improving over the course of medical school [24]. The analysis found no significant overall tendency to over- or underestimate students’ abilities. However, overestimation was found to be more common in communication-based, standardised patient encounters than in objective, knowledge-based performance measures. The results emphasize the complexity of self-assessment and the need for more differentiated research, particularly with regard to the influence of self-assessment in relation to ultrasound skills. Similar to the total absence of any assessment, the mere self-assessment of students also misses objective parameters which can be compared and reproducibly measured. Also, it provides limited insight into the didactic effectiveness of the ultrasound course and students’ competencies.

### Multiple-Choice-Questionnaires (MCQ)

MCQs are flexible assessment tools that can be used independently or as a supplement to other examination methods. As with other questionnaire-based testing tools, evaluation can be conducted quickly and requires limited time and personnel resources, making them both cost- and time-effective. Additionally, they can be administered independently of time and place, further enhancing their practicality. Common types of multiple-choice questions are true/false questions and single best-option multiple-choice questions (MCQs) [25]. As a summative form of feedback, MCQs objectively measure the extent of knowledge and enable high comparability and reproducibility. This allows for consistent evaluation standards across different groups of students and at different time points, promoting a high level of comparability. The learning outcomes of different groups of students or the individual learning progress at different time points can be easily recorded and compared by repeating the questionnaire. Moreover, they provide no information about the participants’ confidence in answering the questions, which can be a critical factor in understanding their readiness to apply the knowledge in practice. However, MCQs are only suitable to a limited extent for testing knowledge transfer, such as interpretation skills, as the background of a dynamic and dialogical examination situation and the global condition of the examined patient cannot be reflected in a rigid set of possible answers. Furthermore, writing context-rich MCQs is challenging, and these questions may inadvertently encourage “cueing”, where the examine selects the correct answer based on choosing the best options, but may not have been able to answer the question based on his own knowledge [26]. Despite these limitations, MCQs can be easily combined with other assessment tools and integrated into pre-existing exams, offering a versatile option for educators.

### Case-based assessments

Case-based examinations evaluate the ability to apply acquired knowledge to clinical scenarios and interpret ultrasound images according to individual clinical cases [10]. Thereby, they provide context and enhance the relevance of the learning material. This approach assesses further cognitive abilities than just the mere retrieval of knowledge, promoting translation cognitive competence by encouraging students to apply learned facts to realistic cases. The presentation of clinical ultrasound cases increases the active participation of students and facilitates the maintenance of knowledge and skills [27]. However, case-based assessments can be performed very differently and may be limited to the assessemt of theoretical knowledge. Besides, technical skills, such as handling the ultrasound device, performing examination techniques or optimizing image acquision may be difficult to evaluate. While these assessments are valuable for gauging knowledge application and interpretation, they may not reflect a student’s practical ability to generate ultrasound images.

### Simulators

Different simulators, such as low-expense phantoms or high-fidelity simulators like mannequins, online scenarios, or virtual reality simulators, can recreate realistic clinical cases while providing a safe learning and assessment environment without real-world stressors. Furthermore, the use of simulators facilitates the organization of structured assessments, as it can be difficult to acquire available patients or models [28]. Simulated assessment formats can also be implemented online, which may further decrease organizational efforts and increase accessibility. Pathologies can be easily integrated into these assessments without the need for suitable patients, offering a flexible and scalable solution for education and evaluation. Furthermore, on simulators, mannequins, and phantoms, interventional ultrasound-guided procedures can be practiced and assessed [29]. Standardized simulators increase the reproducibility of practical assessments and reduce the impact of individual influences, such as different physical conditions of the ultrasound model or the patient [30]. Using a special software, computer-based simulators also allow for the tracking of personal progress to give further insights into the learning process [31].

Nevertheless, depending on the type of phantom or simulator, the properties of human tissue can only be imitated to a limited extent. High-fidelity or VR simulators are available, but in many cases they are very cost-intensive, considering that only one student can train or be tested at any one time. Dynamic examinations involving patient movements, a hallmark of real-life ultrasound, are generally not possible with simulators, which limits their scope. Other limitations include the interpersonal interaction with the patient or model and the application of examination techniques like breathing maneuvers to enhance image quality.

### Objective Structured Clinical Examination (OSCE)

Objective structured clinical examinations (OSCE) are frequently used assessment tools in medical education [10, 32]. Structured checklists provide equivalent scenarios for all examinees with a high degree of objectivity. During OSCEs, the same test tool can evaluate image acquisition, optimization, and interpretation skills. The limited number of scripted scenarios increases comparability by minimizing the impact of external factors. Nevertheless, it can still be supplemented by other test tools, such as drawing exercises, the interpretation of ultrasound images, further questions on theoretic knowledge, or an additional qualitative rating of the acquired ultrasound images [10, 33]. Additionally, context-related skills, such as interaction with the patient or interprofessional communication, can be evaluated. With an appropriate organizational effort and rotation plans, the format enables the conducting of numerous examinations in a short time. It is a standardized procedure in many medical curricula and has proven to be compliant with curricular requirements [34]. As an active and practical examination method, it may motivate medical students to invest additional training in preparation for the assessment. Besides the summative character of the OSCE checklists, the students can receive direct feedback, in which the acting patient or model can participate. Here, however, the focus is more on whether a participant is confident in performing the tasks. Nevertheless, the examination stress is usually quite high due to the participants’ sole focus and the time pressure. This stress is further compounded the brief nature of the examination, which places a direct focus on individual performance.

The digitization of OSCE checklists and the digital assignment to the examinee via QR code can streamline the scoring process, as digital protocols can then be evaluated electronically [35]. OSCEs provide selective control of learning success with many test scenarios simultaneously. This flexibility allows for a combination with other assessment tools, further enriching the evaluation process. However, it may be less suitable for assessing individual organ systems since the single components of the test often merge into an overall result. Further, the limited number of tested scenarios may lead to students preparing only for specific skills, potentially neglecting the clinical relevance of other examinations and broader sonographic principles [36]. As a result, they may be less prepared for individual differences in sonographic examination, such as patient-specific variations in “sonogenicity.” In addition, applied scenarios may not sufficiently represent a realistic hospital setting [33]. With many stations and protocols for different examination situations, the OSCE requires a high level of organizational effort and costs. The format requires examiners with specialist knowledge and specific OSCE training. In order to avoid bias, at least two observers should be involved [10]. While multiple assessors enhance objectivity, they also increase the necessary investment in personnel and resources. Additionally, OSCEs require patients or student models, further adding to logistical challenges.

### Direct observation of procedural skills (DOPS)

In contrast to an OSCE, a direct observation of procedural skills (DOPS) takes place primarily in a clinical setting and focuses on assessing technical skills. Repeated assessments or measurements over a more extended period provide more realistic assessments of competence compared with the typical OSCE, which usually occurs at a set point in time [10]. This approach reduces the need for multiple assessment stations and creates a less stressful examination situation for students. A specific task is broken down into its components, which are evaluated individually.

In the clinical setting, the examiner can be seen as an observer in the background so that the focus is on the interaction with the patient [37]. Unlike the OSCE, DOPS emphasizes clinical behavior and medical decision-making, offering a more dynamic and context-related assessment format. Additionally, DOPS combines learning, supervision, rating, and summative and/or formative feedback, making it an effective tool for evaluating procedural skills.

Especially after gathering first clinical experiences, DOPS is the most effective and widely used tool to assess the acquisition of ultrasound images and examination techniques. Students perform examinations in front of a supervisor, who then provides immediate feedback, combining evaluation with real-time learning. However, as DOPS is a workplace-based evaluation tool, the realistic implementation of DOPS in an undergraduate setting is questionable. Moreover, DOPS requires specially trained examiners and the availability of appropriate patients. A significant limitation of DOPS is that the same patient cannot usually be examined multiple times, reducing reproducibility. Despite these challenges, the method effectively integrates assessment with ongoing clinical practice and learning, making it a valuable tool for procedural skill evaluation.

### Objective structured assessment of ultrasound skills (OSAUS)

The protocol was developed to establish an international and interdisciplinary consensus on ultrasound assessment and was formulated through a Delphi process. It is designed for applicability across various clinical areas and disciplines, enabling an examinee to determine the indication for the examination and to elucidate how it could aid in decision-making and treatment [38]. The OSAUS protocol is a comparable assessment form for ultrasound training across different medical specialties, offering a standardized and approved framework for evaluation. Seven essential sub-steps of an ultrasound examination that should be assessed were identified. For each of these core components, the rater can assign one to five points depending on the learner’s performance, thereby assessing the quality of the examination. This scoring system promotes medical decision-making and the interpretation of ultrasound findings, helping learners connect the diagnostic process with actionable clinical steps.The OSAUS protocol compromises the decision regarding the indication and extension of the ultrasound examination, a systematic approach during the examination, familiarity with ultrasound equipment and its functions, image optimization, interpretation and documentation of the examination and potential findings, and final medical decision-making based on the results of the examination [38]. The protocol is versatile and can be easily applied to different training formats, including courses for various organ systems or clinical specialties. It is also time-efficient, thanks to its universal application across specializations and organs. This generality allows it to serve as a unified examination format for ultrasound training courses across all medical specialties. Increasing the comparability of competence assessments achieved through differing course concepts may facilitate the implementation of a uniform certification process.

However, the protocol has some limitations. Its interdisciplinary creation and application across multiple specialties reduce its specificity, making it less effective for evaluating complex or highly specialized ultrasound examinations.Consequently, the protocol may not adequately assess performance in these cases but further studies are needed to evaluate the relevance of this limitation.

Additionally, OSAUS requires patients or student models, and achieving high levels of objectivity often necessitates multiple assessors, increasing costs and organizational effort. Despite these challenges, it provides an orientation for the development of other assessment tools, such as DOPS and OSCEs.

In the future, undergraduate ultrasound assessment formats are likely to evolve with the integration of both traditional and innovative approaches.

## Summary

In conclusion, several assessment methods are available to measure medical students’ learning success in ultrasound education. Considering ultrasound as a practical skill and applying Miller’s conclusions to medical ultrasound education, the aim must be to provide professional teaching and subsequent assessment at the highest possible level. This includes practical assessment of acquired ultrasound skills in clinical settings. However, this is usually not feasible due to the high number of medical students and the limited availability of suitable patients. Considering the aim of student ultrasound training, which is to prepare students with fundamental knowledge of examination techniques, image acquisition, and interpretation, assessing healthy models in simulated scenarios with classic cases from everyday clinical practice offers a realistic and effective approach to clinical preparation. Several test formats currently used in ultrasound training are all viable options with advantages and disadvantages. The choice of assessment method should be based on the learning objectives and competencies to be assessed, as well as the resources available and the preferences of the educators. Combining different assessment methods may be the most effective approach to evaluate the students’ ultrasound skills and knowledge comprehensively. Following the WFUMB recommendations of structured assessment tools that also consider technical aspects [3], OSCE and DOPS represent the gold standard according to the current state of research and allow a precise examination of learning success while offering high educational value (Table 1).

**Table 1.**
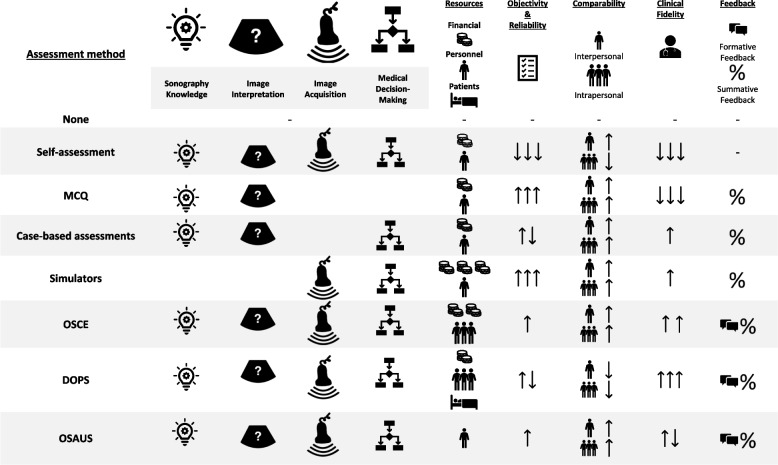
Comparison of various assessment formats based on key criteria essential for conception and implementation

## Role of certification in SUSE

Certification can serve as a quality assurance process as part of professional quality management. It can focus on individuals and their qualifications, processes, standards, facilities, equipment, or training programs. Course and department certifications are widely used in medicine and medical education, respectively, as an external quality indicator. As ultrasound technology becomes increasingly integral to a myriad of diagnostic and therapeutic procedures, certification establishes a standardized measure of skill and knowledge that students must attain. By setting a benchmark for educational attainment, certification helps to guarantee that all medical graduates possess a fundamental level of ultrasound expertise, which is essential for patient safety and effective clinical practice.

Moreover, certification acts as a gatekeeper, ensuring that only those who have demonstrated the requisite understanding and ability in ultrasound use can perform these procedures. This function of certification is not unique to undergraduate medical education, but is also central to established models in countries where sonography constitutes a distinct professional field. International frameworks frequently delineate clearly tiered levels of expertise and incorporate certification as a formalized mechanism to ensure competence and facilitate lifelong learning, and are further reinforced by interprofessional educational models that enhance collaboration across disciplinary boundaries and strengthen alignment between certification standards and clinical roles [39].This is particularly important given the risks associated with incorrect ultrasound interpretation and application, which can lead to misdiagnosis or inappropriate treatment plans. In educational settings, the certification option generates student interest in the method and motivates them to reach and maintain high ultrasound practice standards. It encourages a rigorous learning process and fosters a commitment to continuous improvement and lifelong learning. The certification also provides a clear pathway for students to follow, guiding their studies and practical training in a focused and structured manner. The desire for a uniform regulation or recommendation for student ultrasound training has been increasingly expressed by students for several years. Recommendations on the content of student training have been published by the European Federation of Societies for ultrasound in Medicine and Biology (EFSUMB) [40, 41]. As the interinstitutional certificate is still pending, individual teaching institutions are proceeding with institution-related qualifications, such as certificates and diplomas, that describe assessed knowledge and teaching content according to their curricula. Whether such certifications will lead to increased recognition and possibly to an increased transfer of ultrasound training into medical curricula remains elusive. There are potential positive and negative aspects of an ultrasound certification process in student ultrasound education.

On the one hand, establishing and implementing an appropriate certification can define core competencies, potentially increasing the standardization of ultrasound training. Standardized learning objectives could then support a more comprehensive integration of ultrasound into medical curricula. As ultrasound education heavily relies on the volunteering of student ultrasound tutors in the context of peer teaching, a certification would lead to a higher acceptance and appreciation of their work, which is essential as an incentive to acquire sufficient student tutors to maintain ultrasound education. Furthermore, by creating an official certificate connection to a professional society, an active involvement in ultrasound societies and ultrasound-related research could be strengthened. As an imaging tool where accuracy depends on the examiner’s skills, ongoing practice, acquiring fundamental skills during medical school could relieve overcrowded residency curricula and facilitate career entry. A recognized certification of ultrasound skills upon the end of medical school would ensure the existence of fundamental skills among junior residents and open up more time and resources for specialized training as part of residency training.

On the other hand, the planning and implementation of a certificate, as well as the ongoing control and awarding, would require a significant investment of financial and personal resources and could probably also lead to more bureaucracy in the long term. To be certified, it may be required to complete specific training programs, which can mean extra costs for the participants, especially at the beginning of a career. The potential benefits of a certification can be challenging to predict. Furthermore, if certain institutions wish their ultrasound education to remain extracurricular, it may not be easy to maintain sufficient funding for ultrasound devices and staff.

In conclusion, certification for student ultrasound training has the potential to enhance the quality of training. When officially recognized and broadly accepted, it can function as an incentive for local universities to adapt their curricula to align with the required standards. Steps towards this aim can be the standardization of the criteria for recognizing student ultrasound tutors and the definition of the content of a student ultrasound curriculum. At the same time, a certification would counteract differing levels of ultrasound skills among graduating students. Furthermore, the retention of acquired ultrasound skills should be tested and reinforced over appropriate periods with re-accreditation examinations, which can be continued during residency training. A certificate could help accept and validate ultrasound qualifications already acquired during medical school and facilitate an earlier independent application of ultrasound in residency [42].

## Discussion

Current training structures, course content, and approaches in student ultrasound training are very heterogeneous [14]. This variability becomes even more pronounced when considered within an international framework. In several countries—such as the United Kingdom, Australia, and Canada—sonography is recognized as an autonomous profession, underpinned by profession-specific educational trajectories and formal certification mechanisms [43, 44]. In the UK, for instance, the absence of statutory regulation has led to the establishment of voluntary registration systems and intensified discourse on the need for coherent career progression models and professional legitimization [45]. These developments underscore how national healthcare structures and regulatory frameworks shape both the delivery and professionalization of ultrasound education, offering valuable implications for the refinement of undergraduate training strategies.

However, the assessment of competencies is necessary to ensure a minimum level of competence in ultrasound for the benefit of safe use on patients in terms of the best possible patient care [3]. There are various methods for testing ultrasound knowledge and skills taught in training units, and the different test procedures have certain advantages and disadvantages as state above. The selection of suitable test tools should be based on the purpose of the assessment. This also applies to the timing of the tests. Suppose the assessment is not solely to ensure the learners’ competence but also to measure the effectiveness of the teaching intervention, an additional assessment should be conducted before the training to measure any pre-existing experiences. Otherwise, the assumed effectiveness of the didactic approach would be biased.

Furthermore, the maintenance of ultrasound skills should be assessed to ensure long-term competence. The curricular implementation of ultrasound training should be longitudinal with the continuous aim to re-inforce and connect skill acquaintance and knowledge during medical studies within the different subspecialities over time. In general, the combination of different test procedures allows for the obtainment of a realistic picture of the competencies of the student being tested. The additional collection of different perspectives within evaluation forms is essential for further developing course programs in SUSE [46]. DOPS tests the highest level of a learning process by directly observing the application of newly acquired skills and their influence on the student’s general behavior in clinical situations. Despite organizational difficulties, this assessment form should be pursued when possible.

A valid assessment is the prerequisite for subsequent certification of the tested skills. A universally accepted certification could lead to greater appreciation and recognition of student ultrasound tutors, most of whom work voluntarily. Certification that would also be recognized after graduation could create additional incentives to take an interest in ultrasound and begin to learn it earlier. For the value of such a certificate, it is also important to define which skills have already been sufficiently acquired to be credited for future courses. In addition, in the design of ultrasound courses, the orientation towards uniform learning objectives for obtaining a certificate can promote the standardization of ultrasound teaching. The required investment and potential increase in bureaucracy must be considered both when creating a certification system and when carrying out assessments to achieve a sustainable compromise between feasibility and quality in line with available resources.

This study has several limitations. Although a comprehensive literature search was conducted, no systematic approach was followed, and due to the dynamic publication situation in the field of ultrasound education, it is possible that relevant literature remained to be not included in this review. The intergenerational and interdisciplinary exchange among international ultrasound educators, clinicians, and students, upon which this paper is based, makes it particularly valuable. However, most of the participants are from Germany, meaning the results may be influenced by a European perspective. Furthermore, there is unfortunately no balanced distribution across all medical specialties in which ultrasound is highly relevant, meaning that field-specific nuances of examination formats may not have been sufficiently considered. The advantages and disadvantages of the examination formats should be understood as general tendencies rather than definitive conclusions. Ultimately, the individual implementation of an assessment format or a combination of different formats determines the strengths and weaknesses of the testing method.

Nevertheless, several trends can be identified on how assessment formats will potentially will evolve in the future. The integration of technological innovations into assessments may further increase. This includes the enhanced use of simulations, such as virtual reality and advanced ultrasound simulators. Furthemore, case-based assessments and clinical scenarios will adjunct traditional theoretical test methods, integrating theoretical knowledge into practical contexts, fostering clinical decision-making and critical thinking among students. Additionally, the importance of continuous, process-oriented assessment, as supported by formats like DOPS and OSAUS, is expected to grow. These methods facilitate long-term development and provide regular feedback, leading to sustained improvement of practical skills. It is also anticipated that the integration of self-assessment as a supplementary tool will increase, promoting reflection on one’s strengths and weaknesses, though it will likely remain secondary to formal competency validation. Following validation, certification facilitates widespread recognition. As part of the broader trend towards establishing universally applicable standards for both medical practice and education, this could represent a step towards the standardization of ultrasound competencies acquired during medical school.

## Conclusion

An objective assessment forms the basis for a valid certification system that students, physicians, and patients can trust in. By testing sustainable changes in behavior, DOPS should be prioritized when selecting a suitable examination format. Pre-testing and a review of long-term retention should also be considered, as well as the balance between theoretical and practical assessment forms according to available resources. The combination of different examination modalities makes it possible to obtain an assessment of performance that is as valid as possible. Despite the additional bureaucratic and financial efforts required to implement a certification system, certifications could lead to future maintenance, further implementation and standardization of SUSE, and quality assurance of minimum competencies in the long term.

## Data Availability

Data availability statement: This review article is based on an analysis and synthesis of previously published studies and does not involve the generation of new datasets. All data supporting the findings of this review are available within the referenced articles and publications. No new data were created or analyzed in this study.
